# Characterization and In Vivo Assay of Allantoin-Enriched Pectin Hydrogel for the Treatment of Skin Wounds

**DOI:** 10.3390/ijms24087377

**Published:** 2023-04-17

**Authors:** Rosa Alicia Saucedo-Acuña, Karen Zulema Meza-Valle, Juan Carlos Cuevas-González, Elsa Gabriela Ordoñez-Casanova, Manuel Iván Castellanos-García, Erasto Armando Zaragoza-Contreras, Genaro Federico Tamayo-Pérez

**Affiliations:** 1 Institute of Biomedical Sciences, Autonomous University of Ciudad Juarez, Av. Benjamín Franklin No. 4650, Zona Pronaf Condominio La Plata, Ciudad Juárez C.P. 32310, Mexico; 2 Institute of Engineering and Technology, Autonomous University of Ciudad Juarez, Av. del Charro y Henry Dunan s/n, Omega, Ciudad Juárez C.P. 32584, Mexico; 3 Centro de Investigación en Materiales Avanzados, S.C. Miguel de Cervantes No. 120, Complejo Industrial Chihuahua, Chihuahua C.P. 31136, Mexico; 4 Jefatura de Investigación, Hospital Ángeles Ciudad Juárez, Av. Campos Eliseos 9371, Campos Elíseos, Ciudad Juárez C.P. 32472, Mexico

**Keywords:** pectin, allantoin, hydrogel, skin regeneration, wound healing

## Abstract

This work describes a liquid allantoin-enriched pectin hydrogel with hydrophilic behavior that is supported by the presence of functional groups related to healing efficacy. A topical study shows the effect of the hydrogel application on surgically induced skin wound healing in a rat model. Contact angle measurements confirm hydrophilic behavior (11.37°), while Fourier-transform infrared spectroscopy indicates the presence of functional groups related to the healing effectiveness (carboxylic acid and amine groups). Allantoin is distributed on the surface and inside the amorphous pectin hydrogel surrounded by a heterogeneous distribution of pores. This promotes wound drying with better interaction between the hydrogel and cells involved in the wound healing process. An experimental study with female *Wistar* rats indicates that the hydrogel improves wound contraction, reducing around 71.43% of the total healing time and reaching total wound closure in 15 days.

## 1. Introduction

Skin is the largest organ in the human body. It is in charge of performing important roles such as protection, thermoregulation, and immunological functions [1]. A lack of its integrity is generally referred to as a wound. A chronic wound is a term used to refer to unsuccessful tissue closure. Under this condition, traditional dressings do not cover all the needs for chronic wounds, i.e., a moist environment to promote wound epithelialization or to avoid the adherence of contaminants to prevent infections [2]. In trying to meet the needs for the management of chronic wounds, the development of biocompatible polymeric hydrogels and xerogels began [3,4]. Polymeric hydrogels are three-dimensional networks with high-water content [5], while polymeric xerogels are obtained by slow drying of hydrogels with the advantage of preserving part or all of their porosity [6].

Synthetic and natural polymers have been widely studied for the development of hydrogels or xerogels for wound healing. Synthetic polymers such as poly(ethylene glycol), poly(vinyl alcohol) poly(glycolic acid), poly(lactic-co-glycolic acid), and poly(caprolactone) [7,8,9] and natural polysaccharide- and protein-based polymers such as cellulose, chitosan, collagen, alginate, hyaluronic acid, or pectin have been approved by US Food and Drug Administration (FDA) for medical applications [10,11,12,13].

Reports on cellulose, chitosan, collagen, and pectin show the acceleration in the formation of new cellular mass and extracellular matrix [7,14,15]. Some of the essential needs for wound healing are non-alteration of the tissue repair phases due to gel presence and control of wound exudates due to porosity [7,14,16].

In recent years, interest in the use of pectin hydrogels or xerogels for biomedical applications continues to grow due to the advantages of their biocompatibility, swelling kinetics, and release properties [15,17,18,19,20,21,22,23,24]. Both kinds of gels have been enriched with natural extracts for antibacterial [1], in vivo immunomodulating effects [19], drug delivery [4,6], tissue engineering [15,20,25,26], and wound dressing applications [4,26,27,28,29].

Pectin hydrogels have a three-dimensional structure with the ability to retain moisture and oxygen permeability [1,25,28,29]. Pectin is a biocompatible and nontoxic heterogeneous polysaccharide of vegetable origin widely used in pharmaceutical products [15,18,19,20,21,22,23]. The presence of D-galacturonic acid and methyl ester linked via glycosidic bonds is a guarantee of high solubility, which makes pectin an excellent medium to deliver allantoin in a wound [30,31]. Due to its low antibacterial activity, pectin has been enriched with silver (Ag) or zinc oxide (ZnO) nanoparticles with promising results, but because of its low cost, pectin enrichment with plant extracts is recommended [32]. It should be noted that different types of hydrogels with natural antibacterial extracts have been used in bandages due to their biocompatibility and availability [33]. Therefore, hydrogels obtained from natural polymers would maximize this advantage. Pectin is a polysaccharide with many hydroxyl groups; so, the large number of intermolecular hydrogen bonds contributes to hydrogel adhesion. The homogeneous distribution of allantoin in hydrogel improves its adhesiveness and its antibacterial property to promote wound healing [33,34]. The presence of plasticizer in membranes or hydrogels improves elasticity. One of the main non-volatile liquid plasticizers used for this goal is glycerol. The presence of plasticizer improves the elasticity of the pectin membrane or hydrogel. One of the main non-volatile liquid plasticizers is glycerol. Recent studies showed that the addition of glycerol to chitosan promotes more homogeneous surfaces and increases wettability and flexibility, without significant changes in their chemical structure [35]. Due to the use of hydrogels or membranes over an injured area, infections may occur, mainly when bedsores and necrotic tissues are present [35]. The incorporation of allantoin into pectin hydrogel to stimulate healing promotes necrotic tissue removal ability, cell mitosis, and epithelial cell-stimulation [36], with the advantage that allantoin is soluble in hot water, slightly soluble in cold water and glycerin, and very slightly soluble in alcohol [37].

From vegetable origin, allantoin is one of the most important components of *Aloe vera* and *Calendula officinalis*. It is widely used for tissue regeneration and wound healing [38,39] due to its biocompatibility [34,36], anti-inflammatory and anti-nociceptive effects [40,41,42], and anti-secretory and gastroprotective activity [42]. Further, recently, in vitro and in vivo assays with allantoin alone or in combination with other natural extracts confirm its potential in nerve regeneration [43,44], antibacterial activity [38], and anti-fungal effects against multidrug-resistant yeasts [45].

The development of a natural film based on pectin/allantoin xerogel for wound healing was recently reported as a natural band-aid in the treatment of skin wounds, which favors the development of natural scab by the organism [4]. The aim was to obtain a dry natural film to cover the wound such as an artificial scab, reducing the risk of infection and promoting wound healing. Further, Meza-Valle et al. [31] tested a leading commercial wound healing product based on fresh *Aloe vera* hydrogel, which showed a very similar healing time (29%) compared to that of the natural film of pectin/allantoin with a reduction of 25% [4,31]. That was unexpected because the pectin/allantoin hydrogel was used only during the first 7 days of the animal trial before the natural scab covered the wounds on Day 8 [4]. While for fresh *Aloe vera* hydrogel, an equivalent result was found after 21 days [31]. This finding stimulated our interest in optimizing said film to cover the wound, even over the scab, to enhance wound contraction. Consequently, this work aimed to obtain a pectin/allantoin hydrogel (in the liquid form) to compare the results with the use of the dry film of the pectin/allantoin gel [4]. Thus, the effect of the liquid hydrogel was verified with an animal test, dripping the hydrogel onto the wound during the healing process.

## 2. Results

### 2.1. Pectin Hydrogel Enriched with Allantoin

The pectin hydrogel, obtained by crosslinking with glutaraldehyde, enriched with allantoin and plasticized with glycerol, is a homogeneous liquid. The objective of this system is to speed up the healing process by continuing to cover the wound with the product even after the presence of the natural scab.

**Figure 1:**
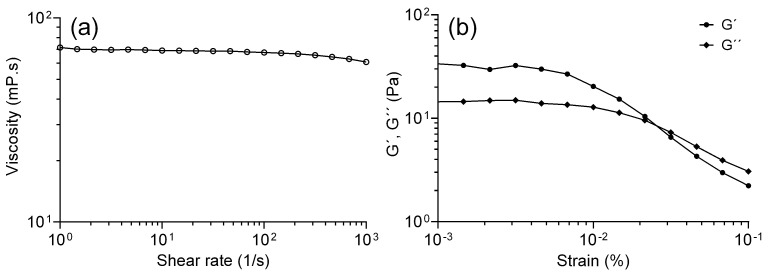
(**a**) Viscosity curves and (**b**) elastic (G′) and viscous modulus (G″) of the pectin−based hydrogel enriched with allantoin.

In hydrogels intended for the development of wound dressings, dynamic rheological/mechanical analysis is very important, because viscoelastic properties provide information about the internal network structure of the dressing material [25,46,47]. Figure 1a shows the viscosity curve regarding the shear rate. The evaluated sample has a Newtonian behavior up to 100 s^−1^ since the viscosity remains practically constant with the increase in the shear rate (67.8 mPa.s). From 100 s^−1^, viscosity exhibits a slight decrease with increasing shear rate, presenting the typical behavior of a pseudoplastic fluid.

Figure 1b shows the deformation scan of the hydrogel. The elastic modulus (G′) at 0.001% was 33.5 Pa and the yield stress or end of the linear viscoelastic region (RVL) was presented at 0.00316%. As noted, G′, related to the hydrogel ability to return to its original position when the deformation force (stress) is removed, dominates over the viscous modulus (G″), related to the energy lost after each deformation cycle. So, the behavior of the evaluated samples is that of a viscoelastic solid (gel). In the case of hydrogels for wound dressing, the value of the G′ should be greater than G”, indicating stable gel structure (network); because if G″ is greater than G′ with increasing strain, it indicates the destruction of the hydrogel network [25,46]. Based on this, the hydrogel will present a structural stability that will not help present phase separation or sedimentation of the components.

### 2.2. FT-IR Analysis

**Figure 2:**
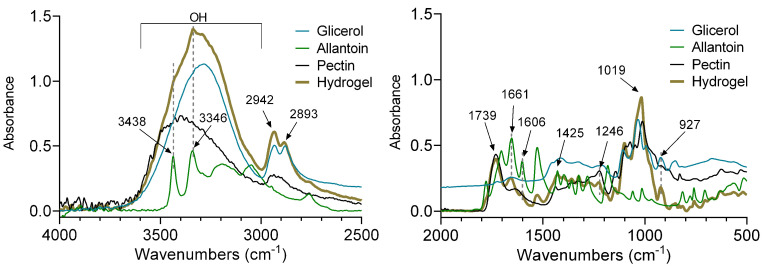
Infrared spectrum of the pectin-allantoin enriched hydrogel. Spectra of pectin, allantoin, and glycerol are included for comparison purposes. Black arrow = characteristic peaks related with functional groups of components.

FT-IR analysis confirms a physical crosslinking of the product, since the spectrum of the hydrogel does not exhibit any chemical interaction among its components (Figure 2). The characteristic wide band from 3600 to 3000 cm^−1^ is assigned to stretching vibrations of the O-H bonds of pectin and glycerol, and the pair of absorptions at 2942 and 2893 cm^−1^ are ascribed to asymmetrical and symmetrical stretching of C-H bonds of glycerol, respectively [35]. Peaks related to allantoin appear at 3438 and 3346 cm^−1^ related to the asymmetric and symmetric stretching vibrations of N-H bonds, respectively [34,36]. Further, the signal at 1739 cm^−1^ is attributed to stretching of the C=O bond of amide (this band is shared with ester groups of pectin). The peaks at 1661 cm^−1^, attributed to C=O on the ring, and at 1606 cm^−1^, ascribed to bending vibrations of the N-H bond, evidence the amide groups. Further, the signal at 1019 cm^−1^ is designed to the C-N bond of amide I [34,35,36]. On the other hand, signals for pectin are observed at 1739 cm^−1^ that is related to the stretching vibration of C=O of the methyl ester groups [48,49,50]. Besides, the peaks at 1425 cm^−1^ and 927 cm^−1^ are ascribed to the bending vibration of the O-H bond, and at 1246 cm^−1^, appears the absorption corresponding to the stretching vibration of the C-O bond of the carboxyl group [48,49,50,51,52,53].

### 2.3. Contact Angle

**Figure 3:**
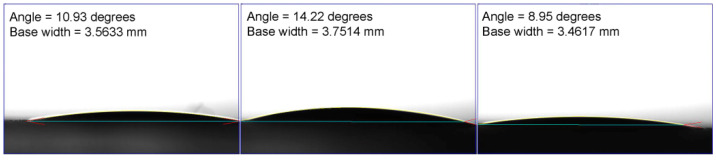
Contact angle of pectin hydrogel enriched with allantoin.

A factor with special consideration in the design of materials for wound healing is the exposure time of the surface with the surrounding biological cells or fluids. Surface characteristics have a direct impact on the biocompatibility of the material, especially wettability, surface chemistry, and surface topography [54]. Figure 3 shows an average of 11.37° of CA obtained from dried hydrogel. This result exhibits the material’s hydrophilicity because of the presence of polar functional groups, predominantly from pectin and allantoin. CA values below 90 degrees are related to high wetting or hydrophilic character because of the presence of polar groups, H-bonding, and hydrophilic groups (hydroxides, carboxylic acids, etc.) [55,56]. Polarity is a crucial property of tissue regeneration, where it is important to allow the flow of body fluids, clotting factors, and inflammatory cells, essentials for wound healing [57,58,59,60,61,62]. In addition, wound healing coatings need to provide a good adhesion and wettability on the surface of the hydrogel, which is related to a good adhesion of the wound [54,63].

### 2.4. Surface Analysis

**Figure 4:**
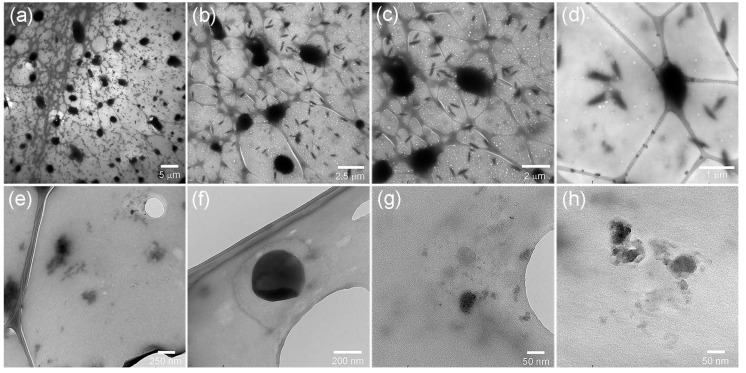
SEM images of the dried hydrogel at different magnifications. (**a**–**c**) Images of the surface showing a homogeneous distribution of porous with the presence of elongated and agglomerated particles of allantoin in the matrix and on the surface, (**d**,**e**) magnifications of elongated and agglomerated particles of allantoin in the matrix, (**f**–**h**) magnifications of agglomerated particles of allantoin on the surface.

Figure 4 shows SEM images of the dried product. Microscopy images show two phases. A continuous phase, corresponding to the pectin matrix, and a dispersed phase, made up of elongated allantoin particles (dark particles) with homogeneous dimensions. Allantoin particles are observed within the film and on the surface. Figure 4a–d show elongated particles of allantoin and some agglomerates in the matrix and on the surface of pectin; whereas Figure 4e,g,h exhibit some irregular agglomerates of allantoin in the matrix, and in Figure 4f, particles of allantoin are observed within a big black dot. The regular distribution of micro and nanopores in the continuous phase should be noted. Such porosity will contribute to the slow draining of a large exudate wound volume and provide a relatively humid environment [3,4,28,33]. This feature also increases nutrient distribution and gas exchange, promoting wound healing [32].

### 2.5. Healing Process

**Figure 5:**
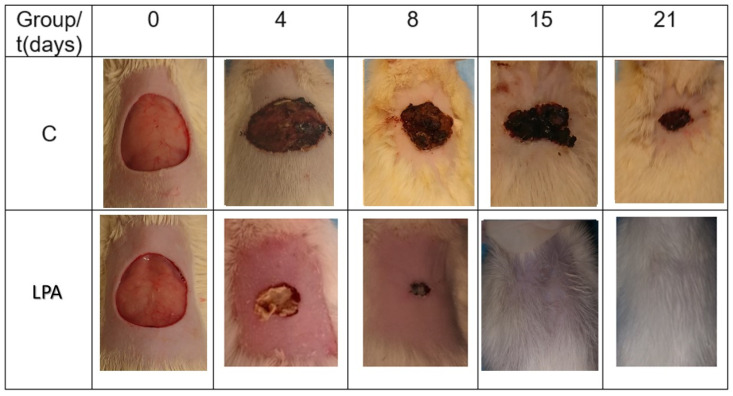
Wound evolution during the healing process.

Table 1 and Figure 5 portray the reduction area of the control group (C) and the group treated with the liquid pectin/allantoin product (LPA) on different days of the treatment. As observed, the wound showed a significant advantage in area reduction from Day 4, when almost 50% of the wound had closed. For the pectin/allantoin film, in the previous study, an equivalent effect was reached up to Day 6 [4]. Note in Figure 5 that the presence of the hydrogel at the wound site does not promote a negative response in the surrounding tissue.

It is worth saying that this new study is taking the control group reported by our group in 2020 [4]. For this, in the new study, four specimens were also studied for the control group for Days 4, 8, 15, and 21, as seen in Table 1. This procedure has been based on: (a) the Animal Research Review Panel (ARRP Webinar Series 2022) to promote the correct analysis of data on the use of animals in research and (b) the principle of 3Rs, Replacement, Reduction, and Refinement of animals in research [64]. Therefore, the data of the new control group in 2022, seen in Table 2, show the statistical analysis between Group C reported in 2020 (C_2020_), where three biopsies were taken for each day (0, 4, 8, 15, and 21) for a total of 15 samples [4]. In this new study, for the LPA group in 2022, 15 samples were also taken on the same days (0, 4, 8, 15, and 21).

Table 3 shows the results of the microscopic analysis of the wound on Days 4, 8, 15, and 21. The hydrogel´s porosity should provide the necessary diffusion of oxygen and humidity without a negative effect or response with the biological surroundings, offering protection at the wound site [32]. The phases of wound healing are divided into inflammation, deterioration, and maturation, although some authors describe it with some intermediate phases, mainly those that overlap each other [7,14]. Inflammation implies coagulation, vasoconstriction, and fibrin formation. Proliferation implies re-epithelialization, neovascularization of blood vessels (angiogenesis), and granulation tissue. Finally, maturation implies the reorganization of the extracellular matrix, degradation, and resynthetization [7]. According to the results in Table 3, the evaluation of the histological parameters shows that the presence of the hydrogel in the wound site does not alter the order of the mentioned phases of the healing process.

**Figure 6:**
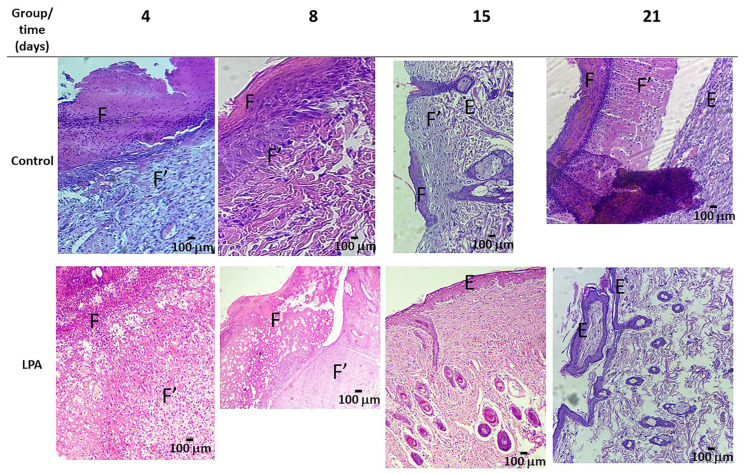
Images of the tissue during the healing process. F = fibrin, F′ = dense fibrous tissue, E = epidermis. Hematoxylin & eosin stain, 10×.

Figure 6 portrays tissue evolution as a function of time (days). On Day 4 of surgery, a diffuse chronic inflammation with the predominance of plasma cells and neutrophils accompanied by the presence of macrophages and fibroblasts was observed. This was observed in both groups, but for LPA, the case of angiogenesis was intense. The absence of epithelium and more abundant production and distribution of fibrin in LPA should be noted. This could be related to the planimetric analysis, where results showed a larger reduction of wound area for this group. On Day 8, both groups showed an abundance of fibrin and inflammation, according to the epithelialization in the areas near the edge of the ulcer; however, important angiogenesis was observed in PLA, while normal angiogenesis was observed in CT. On Day 15, the maturation of the fibrin areas and re-epithelialization of the tissues was noted in both groups, but the LPA Group exhibited an intense epithelialization without inflammation and fibrin, while moderate inflammation was only observed in the CT Group. Finally, on Day 21, CT exhibited a completely reconstructed epithelium under a crust in the process of shedding while the inflammatory cells and fibrin were absent in LPA, showing complete healing.

## 3. Discussion

Natural polymers assure the natural structure and bioactivity, improving the biocompatibility, tridimensional structure, and nontoxicity of new materials for wound healing. Polysaccharides also offer a good similarity to the extracellular matrix (ECM) [7].

Pectin from apple is an anionic heteropolysaccharide, whose carboxyl groups in the chain can be esterified with methyl alcohol, rhamnogalacturonan I, rhamnogalacturonan II, and other complex structures [32]. Until now, a lot of effort has been devoted to adjusting the physical chemistry of chitosan/pectin, which has resulted in higher thermal stability through the addition of glycerol [29]. FTIR showed non-chemical interaction among the components of the hydrogel. This is related to physical crosslinking of the pectin and allantoin hydrogel. Peaks of carboxylic acid and amine groups present in pectin and allantoin confer to the hydrogel a high polarity, an advantage in material adsorption and consequently in wound closure rate [57,58,59,60,61,62].

Biomedical materials based on pectin are widely used for wound dressing. However, due to its poor anti-bacterial activity, pectin has been enriched with silver or zinc oxide nanoparticles with promising results. Nevertheless, pectin enrichment with plant extracts is recommended due to its low cost [32]. It should be noted that several types of hydrogels with natural antibacterial extracts have been used in bandages due to their biocompatibility and availability [33]. Thus, hydrogels obtained from natural polymers would maximize this advantage. Pectin is a polysaccharide rich in hydroxyl groups; consequently, the large number of intermolecular hydrogen bonds contributes to the adhesion of the hydrogel. The homogeneous distribution of allantoin in the hydrogel improves its adhesiveness and its antibacterial property to promote wound healing [33,34].

Soft hydrogels elaborated with water and hydrophilic polymeric networks are naturally hydrophilic [65], but their wetting behavior could turn hydrophobic at high crosslinking because in dense networks there is not enough hydrogen bonding available for the material adhesion [66], or with the presence of additives such as glycerol, the access to these functional groups could be difficult. However, the hydrogel formulation still includes glycerol because its presence facilitates the placement of the hydrogel on the wound. Contact angle measurement confirmed that the hydrophilic character is approximately 60% more for the hydrogel than the dried film of pectin/allantoin [4], which could be related to a more homogeneous distribution of allantoin in the pectin network. The increment in hydrophilicity, even the presence of glycerol, is an advantage because it guarantees the product adhesion to a wound, but with good interaction with proteins and growth factors during wound healing [28,60,61].

SEM showed an amorphous and porous surface, with the abilities of moisture retention and oxygen permeability [1,27,28], where the structure of a natural hydrogel makes possible its use as a support matrix as a connective tissue does [4,28,65,66]. These topographic features are related to the wound healing rate. As observed, wounds treated with the hydrogel show no histological difference; however, clinically, the wound closure took place in approximately a third of the time in relation to the control group, without the presence of abnormal cells. Even so, it is necessary to attend to other parameters and biological conditions.

If a hydrogel for wound healing does not promote the growth of a smooth monolayer of cells on its surface, it would not be considered biocompatible because the material will not enable the growth of tissue on its surface [54,55]. Wettability is one of the most important conditions for this. However, biomaterials exhibiting low CA do not necessarily exhibit enhanced biocompatibility and porosity, as roughness has a great influence too [4,54,67,68]. Petar et al. mentioned that hydroxyethyl cellulose (HEC) with a low CA (36.07°), among sodium chondroitin sulphate (SCS) and hydroxypropyl-methylcellulose (HPMC), exhibited the best properties for in vitro and in vivo trials. Further, Zimina et al. concluded that the increase in the wettability of a porous scaffold based on PLA [68], where its CA decreased from 83.6 ± 1.91° to 62.4 ± 4.17° with the addition of hydroxyapatite, improved the in vivo results. On the other hand, Rezai et al. concluded that surface roughness for PMMA systems was similarly important as low CA values [67].

In our case, comparing the values of CA (18.81°) of pectin–allantoin films [4] and pectin–allantoin hydrogel (11.37°), we suggest that the more homogeneous distribution of pores and allantoin improves the wettability, and consequently the wettability and porosity are very important to enable the percentage size reduction of the wounds during the healing process [4,54,68]. Thus, more research is required to determine how surface properties such as wettability and porosity affect biocompatibility.

The in vivo test confirms that the allantoin-enriched pectin hydrogel exhibited appropriate biocompatibility and pore distribution than the same combination of components in a dry film, with the advantage of better wound healing for the in vivo test, promoting in less time angiogenesis, granulation, and re-epithelialization of the skin. The rate of wound contraction in the group treated with the pectin/allantoin hydrogel is significantly faster than that of the group treated in 2020 with the film of pectin/allantoin with 95% confidence [4]. From Day 4, it was observed that the contraction rate of the wound is higher than the contraction rate of the control group with a positive effect on the healing process. A mathematical equation obtained from SolidWorks^®^ permitted calculating a time of 24 days from the closure of the wound in the control group in 2020 [31], while in the group treated with the allantoin-enriched hydrogel in this work, the wound closure was on Day 15, which means that only in 71.43% of the healing time, the wound treated with pectin/allantoin hydrogel completes the healing process compared to the control group.

It is worth saying that the presence of the hydrogel in the wound site does not alter the order of the phases of the healing process. This is very important because wound healing is an intricate biological mechanism, where the presence of the hydrogel should not alter the sequence of the cellular events to generate new healthy tissue [7]. One of the biggest challenges is the formation of new tissue containing micro-vessels, sweat glands, and follicles. Hence, the development of a wound dressing that inhibits scar formation and promotes hair follicle regeneration is of great clinical importance [7,33]. Previous works reporting in vivo trials with rats with wound dressings based on pectin/allantoin [4] or LMA pectin/calcium (II) [69] obtained similar wound healing results as this work around 21 days after recovery. Similarly, the alginate/pectin film in combination with modified chitosan/curcumin NPs reported comparable results at Day 18 [70], 15 days for *Calendula officinalis* [71], 82% of wound reduction at Day 14 for piperine carbopol hydrogels mixed with *Aloe vera* [72], and for chitosan hydrogels with *Aloe vera*, wounds heal after 13 days [71,72]. Other works, using commercial products such as Restaured^®^, based on *Aloe vera*, showed almost 92% of reduction wound around Day 15 [31], and Debrigel™, based on alginate, sodium carboxymethyl cellulose, and propylene glycol, exhibited 92% of contraction of partial thickness at Day 15; however, this last product was tested in burn wounds [73]. It is worth saying that none of these results report the presence of sweat glands or follicles. Only with the use of proangiogenic peptide nanofiber hydrogels used for skin regeneration in mice, we find the formation of hair around Day 20 [74]. However, the pectin/allantoin hydrogel system began to cover the new skin with hair in the wound area around Day 15.

According to these results, the proposal on the use of a natural band-aid of the pectin/allantoin hydrogel-based film is an option for scratches or to protect wounds to facilitate the mobility of injured people. However, in cases of deep or more extensive skin wounds, hydrogel in liquid form dripped onto the wound should be considered.

## 4. Materials and Methods

### 4.1. Chemicals

Pectin from apple, glutaraldehyde (GA), and hydrochloric acid (HCL) were obtained from Sigma Chemicals, St. Louis, MO, USA; while pharmaceutical grade glycerol and allantoin were purchased from La Corona, S.A. de C.V., Ecatepec, Mexico, and Droguería Cosmopolita, S.A. de C.V., Mexico City, Mexico, respectively.

### 4.2. Synthesis of Pectin Hydrogel Enriched with Allantoin

Pectin hydrogel was obtained from a 5 wt% aqueous solution, using glutaraldehyde (10^−3^ M) as the crosslinker in an acid medium (HCl 10^−1^ M). This solution was kept under mechanical stirring for 2 h at 35 ˚C. The allantoin was then incorporated into the hydrogel in a 1:1 mass ratio and stirring was continued for a further 2 h. Next, glycerol was added as the plasticizer, also in a 1:1 mass ratio regarding the pectin mass, and the system was stirred for another 1 h. Finally, the solution was sterilized with UV and stored in polypropylene Corning Falcon sterile sample containers at standard conditions.

### 4.3. Characterization of the Hydrogel

To avoid water interference during characterization, the hydrogel was dried at 35 °C for 1 week. The functional groups of pectin/allantoin dried hydrogel were examined using an FTIR spectrometer (Alpha-T system, Bruker, Billerica, MA, USA) in the wavenumber range from 4000 to 400 cm^−1^. The surface morphology of the hydrogel was recorded in a scanning electron microscope (JSM-6010Plus, JEOL, Akishima, Japan) under a vacuum of 60 Pa and 15 kV.

The contact angle (CA) was calculated by the sessile drop method using a contact angle meter (FTA-32, First Ten Ånstroms, Portsmouth, VA, USA). The hydrogel sample was deposited in a clean glass sample holder, allowing it to evaporate under laboratory conditions for 24 h. For the measurement, a drop of 5 μL of distilled water was deposited. With the equipment software, the contour of the water drop image was analyzed and the contact angle was determined. The value of the contact angle corresponds to the average of three images.

The rheological properties of the hydrogel were analyzed in a rotational rheometer (MCR 501, Anton Paar, Graz, Austria) at 25 °C, using double gap geometry. The viscosity of the sample was evaluated with respect to the shear speed in a range from 1 s^−1^ to 1000 s^−1^ and its viscoelastic behavior by means of an amplitude sweep using a frequency of 1 Hz.

### 4.4. In Vivo Assay

Sixteen female *Wistar* rats that were 8 weeks old with a weight of 250 g were used for the assay. The assay was previously approved by the Animal Ethics Committee of the Autonomous University of Ciudad Juarez (approval number CIBE-2017-1-45) following the European Animal Research Association (EARA) [75]. Each specimen was placed in a polycarbonate cage at 21 °C and 45% humidity with an ad libitum diet, acclimatizing the animals for 15 days and keeping these conditions for 21 days more [64,76]. A skin wound of 2 cm in diameter was surgically induced on the back of each animal, starting with a scalpel and finishing with scissors. Rats were randomly distributed into two groups: Group C (control) with four animals, where the wounds did not receive any human intervention, and Group LPA (liquid pectin hydrogel enriched with allantoin) with 12 animals, in which the wounds were treated with pectin/allantoin hydrogel. Images of the animals were taken with a comparable scale to obtain a precise measurement of the wound with the SolidWorks (Dassault Systèmes, Vélizy-Villacoublay, France) program. The measurements obtained were used to calculate the rate of wound contraction for both groups. In addition, a histopathological study was carried out to guarantee the quality of the healing tissue between both groups.

### 4.5. Surgical Procedure

Animals were anesthetized intramuscularly in the gluteal region with xylazine (10 mg/kg) and tiletamine/zolazepam (30 mg/kg). Trichotomy of the dorsal spine was performed with a razor and the rest of the hair was removed with Nair^®^ depilatory cream, rinsing with plenty of water and drying with sterile gauze. Surgical excision of 2 cm in diameter was performed in the dorsal area below the shoulder blade. The piece of skin was removed, and the dorsal fascia muscle was exposed. After the surgery and daily from 8 to 20 h, the area exposed was covered for each 2 h with a drop of the pectin/allantoin hydrogel until the 21st day. Even when a scab started to cover the wound, the hydrogel was placed over and on the edge of the wound. Biopsies were performed in a previous assay to compare the results [4], 0 only for Group C and 4, 8, 15, and 21 days for both groups. One rat from Group C and three rats from Group LPA were biopsied each time.

### 4.6. Macroscopic Analysis

**Figure 7:**
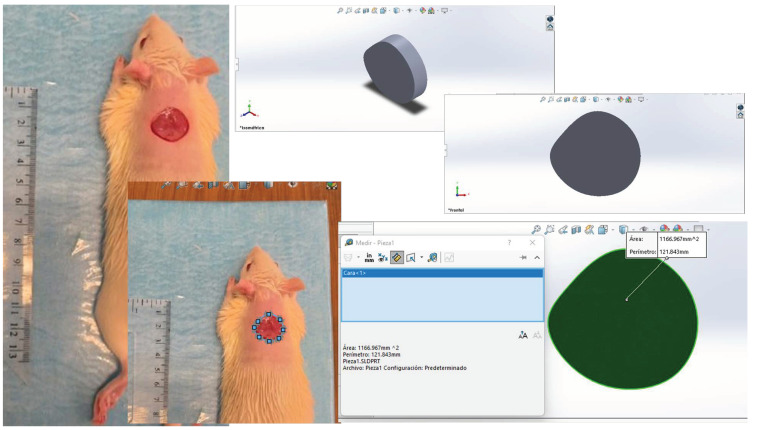
Measurement of the perimeter of the wound and calculation of the area using SolidWorks.

For measurement of wound closure, a photograph was taken while the rats were sedated with isoflurane at 0, 4, 8, 15, and 21 days. After this, each photograph was analyzed by SolidWorks (Figure 7) to compare the size of the area at Day 0 to calculate the wound contraction, using Equation (1).

(1) $$\%AD=\frac{\left(AD_{0}-AD_{t}\right)}{AD_{0}}*100$$

where *AD*_0_ and *AD_t_* refer to the excision area on Day 0 and Day “*t*”, respectively. “*t*” represents 4, 8, 15, and 21 days [4].

### 4.7. Morphology Study

Three rats from Group LPA and one rat from Group C were euthanized by an overdose of anesthesia to take a skin biopsy on Days 4, 8, 15, and 21. Biopsies were taken from a 2 cm elliptical incision around the edges of the wound. Samples were fixed with 10% formalin by 24 h to be dehydrated in graduated series of 80%–100% ethanol solution. The samples were then embedded in paraffin, cut into a 3 μm section, and stained with hematoxylin–eosin. Finally, the samples were evaluated and images were taken using a conventional optical microscope with a light source. Table 4 shows the parameters of evaluation of the slides obtained from the longitudinal sections of the biopsies.

## 5. Conclusions

The hydrogel showed a homogeneous distribution of allantoin with purely physical interactions since the FTIR analysis did not register new functional groups. In addition, the study of the contact angle showed a high hydrophilic character. The amorphous surface and heterogeneous pores make this product an excellent candidate for wound healing, because through said porosities, it would be possible to slowly drain excess moisture from the wound. On the other hand, the use of allantoin-enriched hydrogel reduced healing time by one-third, compared to the control group, even with full recovery of natural hair in the wound area in 15 days. In addition, this hydrogel enriched with allantoin also demonstrated that the delivery of the extract into the wound site improves the wound healing rate.

Due to the known antibacterial properties of allantoin, the antibacterial mechanisms of allantoin in this hydrogel of pectin should be tested, because the development of an adhesive hydrogel with antibacterial properties could solve the treatment of chronic skin wounds with a minimally invasive trial.

## Figures and Tables

**Table 1:** Percentage reduction in wound size during the healing process.

Day	C (%)	LPA (%)
4	21.48	50.87 ± 10.63
8	45.60	70.92 ± 8.51
15	82.85	100 ± 0.00
21	92.82	100 ± 0.00

**Table 2:** Statistical analysis of the percentage size reduction of the wounds during the healing process for C_2020_ and LPA, both made in triplicate.

Day	C_2020_ (%)	LPA (%)	Significance
4	18.48 ± 3.12	50.87 ± 10.63	0.002
8	47.49 ± 3.89	70.92 ± 8.51	0.018
15	83.99 ± 2.15	100 ± 0.00	0.002
21	93.58 ± 3.70	100 ± 0.00	0.025
LSD (least significance difference) test (*n* = 15) for each group test (*p* < 0.05)			

**Table 3:** Evaluation of histological parameters.

Group	t (Days)	Parameter			
		Inflammation	Fibrin	Angiogenesis	Epithelialization
C_T_	4	+++	++	+	−
	8	+++	+++	++	+
	15	++	+++	+++	++
	21	+	++	++	+++
LPA	4	+++	+++	+++	−
	8	+++	+++	+++	+
	15	−	+	+++	+++
	21	−	−	+++	+++

(−) absent, (+) mild, (++) moderate, (+++) intense.

**Table 4:** Parameters for microscopy analysis [31].

Parameter	Scale			
Inflammation	−	+	++	+++
Angiogenesis	−	+	++	+++
Fibrin	−	+	++	+++
Epithelialization	−	+	++	+++
(−) absent, (+) mild, (++) moderate, (+++) intense				

## Data Availability

All data obtained from this study can be found in the Master Program in Chemistry Biology Science and the General Office of Research and Postgraduate, both at the Autonomous University of Ciudad Juarez, and can be requested through the corresponding author.
